# Effects of the Implementation of Transport-Driven Poverty Alleviation Policy on Health Care–Seeking Behavior and Medical Expenditure Among Older People in Rural Areas: Quasi-Experimental Study

**DOI:** 10.2196/49603

**Published:** 2023-11-28

**Authors:** Yuanyang Wu, Qianning Wang, Feiyang Zheng, Tiantian Yu, Yanting Wang, Si Fan, Xinping Zhang, Lianping Yang

**Affiliations:** 1 School of Medicine and Health Management Tongji Medical College Huazhong University of Science and Technology Wuhan China; 2 School of Public Health, Sun Yat-sen University Guangzhou China; 3 Sun Yat-Sen Global Health Institute, Institute of State Governance, Sun Yat-Sen University Guangzhou China; 4 Institute for Global Health and Development, Peking University Beijing China

**Keywords:** transport-driven poverty alleviation, health care–seeking behavior, medical expenditures, difference-in-differences, quasi-experimental study

## Abstract

**Background:**

Improving the rural residents’ accessibility to and affordability of health care is recognized as a common target globally. The Health in All Policies approach, from the Declaration of Helsinki to the United Nations’ *Decade Of Healthy Ageing*, strengthened the far-reaching effect of large-scale public policies on health care–seeking behavior; however, the effects of national transport policy on health care–seeking behavior is unclear.

**Objective:**

This quasi-experimental study aimed to examine the effects of the implementation of transport-driven poverty alleviation (TPA) policy on health care–seeking behavior and medical expenditure among older adults in rural areas and the mechanism underlying these effects.

**Methods:**

We designed a quasi-experiment to estimate the effects of TPA policy implementation on health care–seeking behavior and medical expenditure among older adults in rural areas through a difference-in-differences (DID) analysis based on data from the China Health and Retirement Longitudinal Study in 2011, 2013, 2015, and 2018. The underlying mechanism was analyzed and effect modification patterns were further investigated by poor households, health status, and age.

**Results:**

Our findings validated a positive contribution of TPA policy on health care–seeking behavior among older adults in rural areas. After the implementation of TPA policy, the number of inpatient visits increased by annually 0.35 times per person, outpatient medical expenditure increased by 192% per month, and inpatient medical expenditure increased by 57% annually compared with those of older adults in rural areas without the implementation of TPA policy. Further, there was a significant modification effect, with a positive effect among poor households, healthier older adults, and those aged 60-80 years. Additionally, the policy improved the patients’ capabilities to seek long-distance care (β=23.16, 95% CI –0.99 to 45.31) and high-level hospitals (β=.08, 95% CI –0.02 to 0.13), and increased individual income to acquire more medical services (β=4.57, 95% CI –4.46 to 4.68).

**Conclusions:**

These findings validate the positive contribution of TPA policy on health care–seeking behavior among older adults in rural areas; however, the medical expenditure incurred was also high. Concerted efforts are needed to address health care–seeking dilemmas in rural areas, and attention must be paid to curbing medical expenditure growth for older adults in rural areas during TPA policy implementation.

## Introduction

Poor health care access and high medical expenditure in rural areas are major causes of inequalities and global health burden [1]. Growing inequalities between rural and urban areas in health care use and health outcomes have been reported in globally—for example, rural residents who make up about 80% of the population of 1.4 billion individuals enjoyed about 20% of the national health resources in China—and high-level hospitals are scarce in rural areas [2,3]. Improving the rural residents’ accessibility to and affordability of health care is recognized as a common target in both high-income and transitional counties [4].

It was a milestone that the Health in All Policies (HiAP) approach proposed by the Declaration of Helsinki placed emphasis on social determinants of health, in which transport was recognized as a determinant in health care–seeking behavior by influencing geographic accessibility and spatial behavior [5]. Previous studies paid substantial attention to emergency medical transportation services and road traffic mortality [6,7].

Andersen’s health behavior model pointed out that the use of medical services was affected by individual predisposing characteristics, enabling resources, and needs [8]. Arcury et al [5] further added geographic accessibility and spatial behavior in the health behavior model, including distance, transportation availability, and activity space. The distance decay effect showed that the interaction between the 2 sites decreased with an increase in the distance between them, and this effect of distance was also observed on the use of medical services [9]. Zielinski et al [10] found that patients living further than 40 km of a hospital had lower use of secondary health care services based on the data from the Care Data Warehouse in Östergötland, Sweden [10]. From the perspective of transportation availability, Arcury et al [5], using survey data from a sample of 1059 households located in 12 western North Carolina counties, found that those who had a driver’s license had 2.29-fold more health care visits for chronic care and 1.92-fold more visits for regular checkup care than those without a driver’s license [11]. In terms of activity space, the development of transportation expanded the scope of residents’ activities and facilitated access to remote medical services. Liu et al [12] found that after the opening of high-speed railway, Chinese patients were more likely to go to areas with a high density of health care resources. Rural residents were willing to travel long distances to receive quality services, which was not available locally or when their primary care facilities were located in urban centers [13].

Poor transport infrastructure contributes profoundly to poverty and economic burdens. Taking into account the needs of transport infrastructure in impoverished areas, the Ministry of Transport of China issued the “13th Five-Year Plan for Transport Poverty Alleviation” (hereinafter called “The Plan”), which is committed to strengthening the transport infrastructure in impoverished areas in 26 provinces of China [14]. The Plan aimed to further improve the construction of transport infrastructure in impoverished areas, enhance the capacity and level of transport services, and strengthen the capacity for transport safety and security, with a view to comprehensively resolve the backwardness of transport infrastructure in impoverished areas by 2020. A detailed description of the Transport-driven Poverty Alleviation (TPA) policy is showed in Multimedia Appendix 1.

Better transport has been associated with low transport costs and individual income growth, which are key factors affecting health care–seeking behavior. On the one hand, better transport mitigated the distance decay effect in medical service usage. Time, money, and energy spent for seeking medical treatment were regarded in determining the transport cost, and increased the price of medical services [15]. Alam et al [16] found that transport costs were an important part of medical expenditures by comparing different access to health services during pregnancy, delivery, and the postpartum period among women. In addition to direct economic costs, long-distance travel led to greater pain and discomfort and unexpected physical costs [17]. For example, when the distance to a hospital increased, residents’ health care usage significantly decreased, and the probability of outpatient care also decreased [9]. In contrast, convenient transport generated many social benefits, including lesser travel time and medical expenses and high-quality medical resources.

On the other hand, transport promoted economic development and increased personal income in rural areas due to the fundamental role of transport infrastructure. Income growth in rural areas after the implementation of TPA policy improved the rural residents’ ability to receive health care services, such as higher-income groups in China having a better health status and using more health care services [18].

In summary, high-credibility empirical evidence is needed urgently to implement the HiAP approach to achieve Millennium Development Goals, even though few studies have verified the relationship between transport and health care–seeking behavior. TPA policy was implemented in China, where the fight against poverty made a comprehensive success with 98.99 million poor households, 832 poor counties, and 128,000 poor villages being lifted out of poverty. This study aimed to explore the effect of TPA policy on health care–seeking behavior and medical expenditure among older adults in rural areas by using data from the China Health and Retirement Longitudinal Study (CHARLS).

## Methods

### Study Design

This is a quasi-experimental design study, and the policy’s effect was examined using a difference-in-differences (DID) method with the 2011 and 2018 CHARLS panel data. Differences in health care–seeking behavior and medical expenditure before and after TPA policy implementation were analyzed.

### Participants

For randomization, participants aged 45 years and older were selected randomly from 28 provinces, 150 counties, 450 communities (villages), and 12,400 households. Within each selected household, one resident aged 45 years or older was selected randomly for the sample and their spouse was automatically included. If nobody aged 45 years or older lived in the selected household, the household was skipped. Participants aged 60 years and older or those who had all variables in our study based on the 4 waves of the CHARLS were included.

### Intervention and Control Groups

The intervention was the TPA policy implemented in 2016 [14], which was measured by a dummy variable indicating whether the TPA policy is implemented or not and rural road mileages. The treatment group comprised participants in provinces covered by the policy, such as Yunnan and Xinjiang Provinces, and the control group comprised participants in provinces not covered by the policy, such as Shandong Province (details of the national policy are shown in Multimedia Appendix 1).

### Outcomes

The primary outcomes were health care–seeking behaviors and medical expenditure. Health care–seeking behaviors included the number of outpatient visits in last month and inpatient visits in the last year. Medical expenditure measures included total expenditures for outpatient care in the last month and total expenditures for inpatient care in last year, measured in Chinese Renminbi (Yuan).

### Data Sources

The data were mainly obtained from the 2011, 2013, 2015, and 2018 waves of CHARLS, which is an ongoing prospective cohort study on the determinants of healthy ageing in members of the population aged 45 years and older. The CHARLS was led by the National Development Research Institute of Peking University and jointly executed by the China Social Sciences Survey Center of Peking University and the Youth League Committee of Peking University. Its baseline survey encompassed 450 villages and communities nationwide, with a sample size of 17,708 individuals in 10,257 households. The CHARLS uses a systematic random sampling strategy to select respondents. First, district and county units are implicitly stratified by region, rural or urban areas, and gross domestic product per capita. Based on the probability proportional to population size, 150 district and county units were randomly chosen among all county units, and 3 village or community units were further randomly selected within each county unit. The team developed a special mapping software to help draw a sample frame of all households in each unit. Within each household selected from the mapping frame, 1 resident aged 45 years or older was randomly chosen to be the main respondent, and this person’s spouse was automatically included in the sample. To avoid human error and manipulation, each sampling stage was computerized, and all interviews were conducted using computer-aided personal interview technology [19]. The 4 waves used the same ascertainment and assessment protocols. The rural road mileages measuring TPA policy implementation were obtained from 2011 to 2018 from the *China Transport Statistical Yearbook*.

Information collected mainly included basic sociodemographic status (ie, age, sex, marital status, education, and economic status), health status (ie, self-reported ability to perform basic activities of daily living and the number of chronic diseases), daily behavior habits (ie, smoking and drinking), access to health care services (ie, distance to outpatient or inpatient care, and hospital level of outpatient or inpatient care), health care–seeking behavior (ie, outpatient and inpatient visits), and medical expenditure (ie, outpatient or inpatient expenditure).

The basic sociodemographic status, health status, and daily habit factors were selected as covariates. A detailed definition of covariates is shown in Table 1.

**Table 1:** Characteristics of the participants in the intervention and control groups from 2011 to 2018.

Variables			Total sample		Control group		Intervention group
Age (years), mean (SD)			68.38 (6.89)		68.82 (7.27)		68.28 (6.80)
Sex (male), n (%)			3333 (49.71)		538 (48.59)		2778 (49.65)
Female, n (%)			3372 (50.29)		570 (51.41)		2819 (50.35)
Marital status (married), n (%)			5475 (81.67)		926 (83.60)		4545 (81.22)
Divorced or never married, n (%)			1230 (18.33)		182 (16.40)		1052 (18.79)
Years of education, mean (SD)			4.18 (4.11)		4.01 (4.19)		4.21 (4.09)
Number of chronic diseases, mean (SD)			1.17 (1.31)		1.03 (1.24)		1.20 (1.33)
**Disability status, n (%)**							
	Yes	780 (11.64)		112 (10.16)		668 (11.95)	
	No	5925 (88.36)		996 (89.83)		4929 (88.05)	
**Smoking status, n (%)**							
	Yes	1139 (16.99)		176 (15.90)		965 (17.25)	
	No	5566 (83.01)		932 (84.10)		4632 (82.75)	
**Drinking status, n (%)**							
	Yes	2156 (32.16)		379 (34.26)		1772 (31.66)	
	No	4549 (67.84)		729 (65.74)		3825 (68.34)	
Economic status^a^, mean (SD)			5.26 (3.44)		6.03 (3.53)		5.08 (3.39)
Distance to outpatient care (km), mean (SD)			15.31 (104.93)		10.68 (31.64)		16.11 (112.85)
**Hospital level for outpatients, n (%)**							
	County	5070 (75.62)		760 (68.66)		4337 (77.50)	
	Prefectural	1175 (17.53)		244 (22.05)		912 (16.31)	
	Provincial	460 (6.85)		104 (9.29)		348 (6.19)	
Distance to inpatient care (km), mean (SD)			41.85 (173.93)		27.18 (54.18)		44.42 (187.06)
**Hospital level for inpatients, n (%)**							
	Country	5083 (75.82)		788 (71.17)		4295 (76.75)	
	Prefectural	1186 (17.70)		229 (20.72)		963 (17.21)	
	Provincial	436 (6.39)		91 (8.11)		339 (6.04)	
Individual income (yuan; log-transformed), mean (SD)			2.82 (3.58)		3.07 (3.85)		2.76 (3.51)
Observations, n			6705		1108		5597

^a^Logarithmic value of annual per capita income.

### Statistical Analysis

#### Overview

The effect of TPA policy on health care–seeking behavior, including outpatient visits (model 1) and inpatient visits (model 3), and medical expenditure, including outpatient expenditure (model 2) and inpatient expenditure (model 4), were estimated using 4 DID models [20] in which outpatient and inpatient expenditure were in logarithmic form.

A binary policy indicator variable *treat* was used to decide between intervention and control, and a binary time variable *post* was used to decide between pre- and postintervention. The coefficient of product the terms *treat* and *post* was the policy effect. The specific model was set as follows:

*Healthservice_utilization_it_* = *α*_0_ + *β*_1_*treat_it_* + *β*_2_*post_it_* + *β*_3_*treat* × *post_it_* + *β*_4_*control_it_* + *ε_it_* (1)

where *treat_it_* is the policy indicator dummy variable, indicating that the participant is covered by the TPA policy, during period *t* (the treatment group), valued at 1, and those not covered by TPA policy (the control group), valued at 0. *post_it_* is a time dummy variable, and the value is 1 after issuing the TPA policy (the 2018 wave) and 0 before issuing of the TPA policy (the 2011, 2013, and 2015 waves). *treat_it_ ×* *post_it_* is the product term of *treat_it_* and *post_it_*. *control_it_* is a series of control variables, β_1_, β_2_, β_3_, and β_4_ are the regression coefficients, where β_3_ is the effect of TPA policy, and *ε_it_* is the random error.

The mechanism underlying the effects of TPA policy implementation on health care–seeking behavior and medical expenditure was analyzed using 5 DID models: regression models to determine the association between traveling distance to outpatient visits and TPA policy implementation (model 5), hospital level of outpatient visits and TPA policy implementation (model 6), traveling distance to inpatient care and TPA policy implementation (model 7), hospital level of inpatient visits and TPA policy implementation (model 8), and individual income and TPA policy implementation (model 9).

#### Effect Modification Analysis

To explore possible effect modifications by economic status (ie, poor household: yes vs no), self-reported health status (ie, healthy and unhealthy), and age (ie, around 60-70, 70-80, and ≥80 years), we performed effect modification analysis stratified by these potential modifiers (Multimedia Appendix 2). According to the Andersen’s health behavior model, age was the individual predisposing characteristic, economic status was the enabling resource, and health status was the need factor, which impact health care usage behavior [21,22].

#### Sensitivity Analyses

First, the independent variable of TPA policy implementation was replaced by rural road mileage, and the effects of rural road mileage on health care–seeking behavior and on medical expenditure were examined by using a fixed-effects model. Second, the validity of the identification assumption of the DID model was verified by temporal trends in outpatient visits, inpatient visits, and expenditure of outpatients and inpatients, respectively, from 2011 to 2018. Simultaneously, a propensity score matching method was used to overcome the differences between the control and intervention groups, and then a weighted DID estimation was performed after matching to adjust the sampling. Finally, the dependent variables were replaced with out-of-pocket medical expenditure to exclude the effect of health insurance, because out-of-pocket medical expenditure was not reimbursed by health insurance. Furthermore, the regression analysis was restricted to older adults without pension to avoid the estimation bias from pensions. We further performed a placebo test to best attempt to overcome interferences from other poverty alleviation policies by adjusting the policy time to 2015.

### Ethical Considerations

The studies involving human participants were reviewed and approved by the Research Ethics Committees of Peking University (IRB00001052-11015) [23]. This survey was anonymous and the answers are protected by privacy laws. Written informed consent clarifying the study purposes was obtained from each participant before completing the interview.

## Results

In total, 6705 participants (mean age 68.38 years; 3333, 49.71% male; 5475, 81.67% married) were included. Of them, 5597 (83.48%) and 1108 (16.52%) participants were in intervention and control groups, respectively. Participants in both groups had similar covariate distributions.

Their mean number of outpatient and inpatient visits was 2.37 (SD 2.66) and 1.57 (SD 1.34) in 1 month and 1 year, respectively. The mean log-transformed outpatient and inpatient expenditures were 5.84 (SD 1.49) and 9.20 (SD 1.28), respectively. Mean inpatient visits increased from 1.47 (SD 1.11) to 1.61 (SD 1.79) in the control group and from 1.53 (SD 1.24) to 1.70 (SD 1.52) in intervention group. Mean inpatient expenditure increased from 9.72 (SD 1.14) to 9.82 (SD 1.31) in the control group and from 8.96 (SD 1.27) to 9.39 (SD 1.23) in treatment group (Multimedia Appendix 3).

Table 2 shows the effect of TPA policy implementation on outpatient and inpatient visits of older adults in rural areas with controlling potential confounders. After the implementation of TPA policy, the number of inpatient visits increased by 0.35 times annually, the outpatient medical expenditure increased by 192% per month, and the inpatient medical expenditure increased by 57% annually among the older adults in rural areas. Other control variables also influenced health care–seeking behavior and medical expenditure. Women had higher outpatient expenditures but lower inpatient visits than men. Higher the prevalence of chronic diseases among older people, greater the number of outpatient visits, outpatient expenditures, inpatient visits, and inpatient expenditures. Similarly, disabled older adults or those with a better economic status had more outpatient and inpatient visits. However, older adults who smoked and drank had lower outpatient expenditures and fewer inpatient visits.

**Table 2:** Regression models to analyze the associations of Transport-driven Policy Alleviation (TPA) policy implementation with health care–seeking behavior and medical expenditure among older adults from 2011 to 2018. All models have time-fixed effects.

Variables	Model 1 (2779 observations; *R*^2^=0.007)		Model 2 (5131 observations; *R*^2^=0.233)		Model 3 (2235 observations; *R*^2^=0.024)		Model 4 (699 observations; *R*^2^=0.075)	
	β (95% CI)	*P* value	β (95% CI)	*P* value	β (95% CI)	*P* value	β (95% CI)	*P* value
TPA	–.11 (–0.38 to 0.15)	.40	1.92 (1.58 to 2.26)	<.001	.35 (0.21 to 0.50)	<.001	.57 (0.34 to 0.79)	<.001
Age	–.003 (–0.02 to 0.01)	.73	.01 (–0.01 to 0.03)	.34	.003 (–0.01 to 0.01)	.45	–.01 (–0.03 to 0.01)	.20
Sex (female)	.04 (–0.22 to 0.31)	.75	.50 (0.20 to 0.80)	<.001	–.18 (–0.34 to 0.03)	.05	–.08 (–0.32 to 0.16)	.52
Marital status (married)	–.05 (–0.31 to 0.21)	.70	–.03 (–0.32 to 0.27)	.87	–.05 (–0.20 to 0.11)	.16	.10 (–0.14 to 0.35)	.41
Years of education	–.02 (–0.05 to 0.01)	.11	.13 (0.09 to 0.16)	<.001	.003 (–0.01 to 0.02)	.48	.02 (–0.01 to 0.04)	.13
Chronic diseases	.08 (0.01 to 0.15)	.03	.02 (–0.06 to 0.10)	.60	.07 (0.03 to 0.11)	.01	.004 (–0.06 to 0.07)	.88
Disability (yes)	.34 (0.04 to 0.63)	.03	.26 (–0.07 to 0.58)	.13	.14 (–0.01 to 0.30)	.01	.50 (0.26 to 0.75)	<.001
Smoking (yes)	–.01 (–0.34 to 0.32)	.95	–.87 (–1.22 to –0.52)	<.001	–.27 (–0.49 to –0.04)	.05	–.21 (–0.60 to 0.18)	.30
Drinking (yes)	–.21 (–0.49 to 0.06)	.13	–.24 (–0.55 to 0.07)	.13	–.20 (–0.37 to –0.03)	.64	–.13 (–0.41 to 0.15)	.36
Economic status^a^	.01 (–0.02 to 0.04)	.61	.05 (0.01 to 0.09)	.01	–.002 (–0.02 to 0.02)	.67	.01 (–0.02 to 0.04)	.34
Cons^b^	2.57 (1.27 to 3.88)	<.001	2.22 (0.73 to 3.72)	.01	1.33 (0.55 to 2.12)	.06	9.15 (7.85 to 10.44)	<.001

^a^Logarithmic value of the annual per capita income.

^b^Cons: constant term.

**Figure 1:**
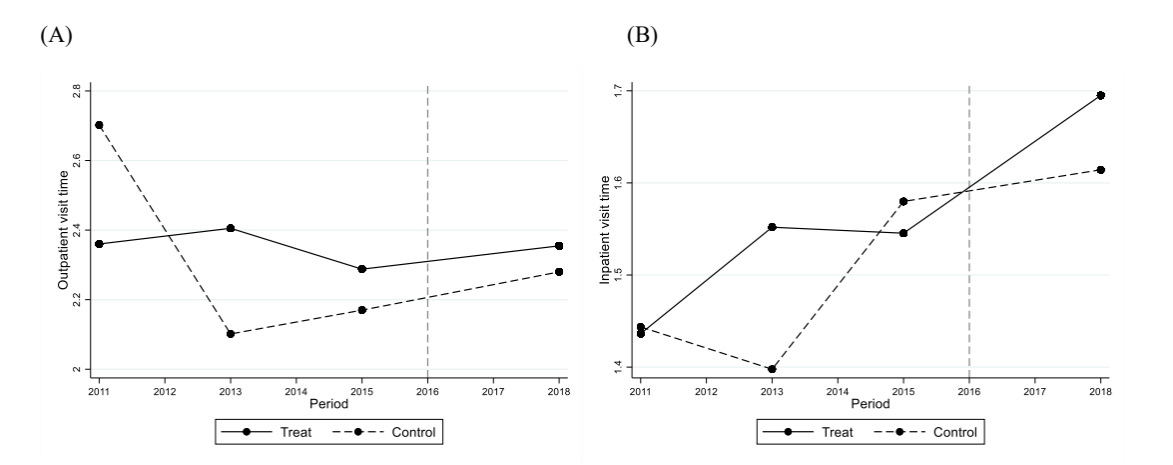
Parallel trend of older adults’ outpatient (A) and inpatient (B) visit times from 2011 to 2018.

**Figure 2:**
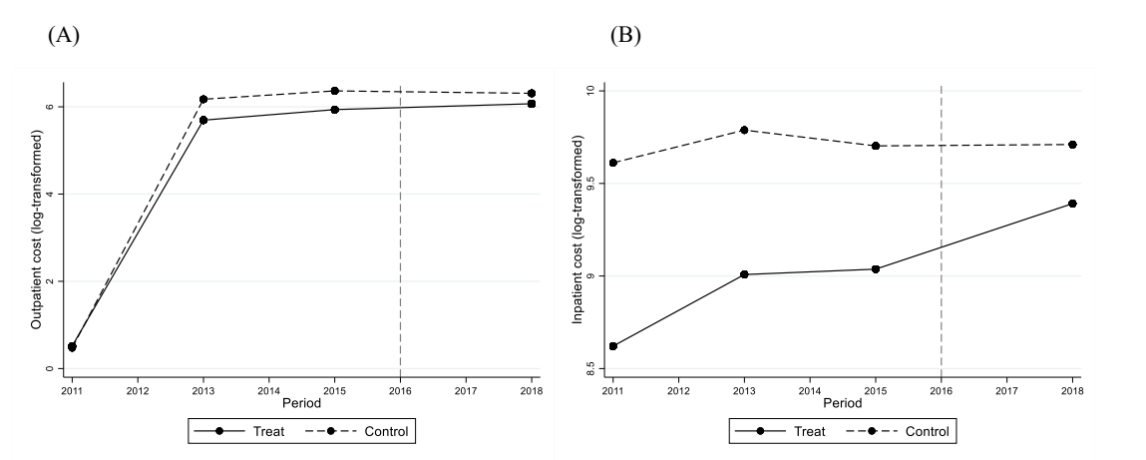
Parallel trend of the older adults’ outpatient (A) and inpatient (B) expenditures from 2011 to 2018.

On sensitivity analyses, first, rural road mileage had a significant positive effect on the number of inpatient visits (*P*<.001) and outpatient expenditure (*P*<.001) for older adults in rural areas, which was generally consistent with results shown in Table 2. Second, Figures 1 and 2 show that outpatient visits, outpatient expenditure, and inpatient expenditure had a similar temporal trend between the intervention and control groups, which verified the validity of the parallel trend assumption. After matching, there was still a positive effect of TPA policy implementation on outpatient and inpatient expenditures (*P*<.001). Third, the estimates of medical expenditure and the outcomes among older adults without pension supported the positive effect of TPA policy implementation on inpatient visits and medical expenditure (Multimedia Appendix 4).

The effects of TPA policy on health care–seeking behavior were also examined and are summarized in Table 3. Models 5-9 show the effect of TPA policy implementation on travelling distance and hospital level of outpatient or inpatient visits. TPA policy implementation significantly increased patients’ traveling distance to hospitals by 23 km compared to that of patients without a TPA policy (*P*=.04). The choice of hospital level was increased by 0.08 points after TPA policy implementation (*P*=.01). Model 9 shows that TPA policy implementation increased individual incomes of older adults in rural areas by 457% (*P*<.001).

**Table 3:** Regression models analyzing the association between Transport-driven Poverty Alleviation (TPA) policy implementation on travelling distance, hospital level, and individual income among the older adults in rural areas from 2011 to 2018. All models had control variables.

Variables	Model 5 (1620 observations)		Model 6 (770 observations)		Model 7 (1788 observations)		Model 8 (1313 observations)		Model 9 (12,600 observations)	
	β (95% CI)	*P* value	β (95% CI)	*P* value	β (95% CI)	*P* value	β (95% CI)	*P* value	β (95% CI)	*P* value
TPA	9.66 (–4.38 to 23.70)	.18	–.01 (–0.08 to 0.06)	.81	23.16 (0.99 to 45.31)	.04	.08 (0.02 to 0.13)	.01	4.57 (4.46 to 4.68)	<.001
Cons^a^	24.678 (–50.36 to 99.71)	.52	1.23 (0.86 to 1.60)	<.001	165.16 (43.14 to 287.19)	.008	1.45 (1.13 to 1.77)	<.001	–1.20 (–1.77 to –0.62)	<.001

^a^Cons: constant term.

The potential effect modifications were examined in subgroups by poor household, self-reported health status, and age and are summarized in Multimedia Appendix 2. In the overall sample, after the implementation of TPA policy, the outpatient expenditure increased by 469% and the inpatient expenditure increased by 85.9% among poor households. In addition, TPA policy implementation increased medical expenditures of older adults with better self-rated health status significantly compared to the medical expenditures of those with poor health. When considering differences in age, TPA policy implementation increased outpatient and inpatient expenditures among 60-80–year-old adults significantly (*P*<.001).

## Discussion

### Principal Findings

In this quasi-experimental study of a nationwide sample of older adults in rural areas, we observed a positive effect of the implementation of a national transport policy on health care–seeking behavior. Empirical results show that TPA policy implementation increased inpatient visits and outpatient and inpatient expenditures significantly. After conducting sensitivity analyses, the promoting effect of TPA policy implementation on inpatient visit times and medical expenditure among older adults in rural areas was still supported.

With rapid growth of the economy, differences in the usage of medical services and health inequality between urban and rural residents in China have been increasingly consequential, and rural residents have been at a disadvantage [24]. In China, due to the disparity between urban and rural areas, medical resources are concentrated in the large urban hospitals, while there are relatively poor resources in rural health care institutions, such as rural and community-level hospitals. Rural residents living in remote areas have faced many limitations when using medical services due to long distances to hospitals and inconvenient transport in rural China. Transport barriers in rural areas may be an important factor affecting medical service usage among older adults in rural areas [25]. When the distance to hospitals increased, residents’ use of health care significantly decreased, and the probability of outpatient care also decreased [26]. Rural patients were more likely to travel longer distances for access to high-quality medical resources when there was a lack of such resources in their areas of residence; therein, the availability of transportation played an important role.

Previous studies have reported the positive role of transport in health care–seeking behavior [11-13,27]. For example, Badji et al [27] found that when public transport availability was high, people with disabilities visited their general practitioners on average 0.5 more times per year without considering the associated medical expenditure. In particular, it was worth noting that TPA policy implementation also increased high-level health care usage, such as inpatient visits in high-level hospitals, which was consistent with Liu et al’s [12] findings that 6.9% of outpatients in the Sichuan province of China were transferred to high-level medical institutions via high-speed rail. Nevertheless, our conclusions were of more universal significance because of a nation-level data analysis and paying attention to medical expenditure in rural areas [11-13,27]. TPA policy implementation not only improved the accessibility of older adults in rural areas to acquire medical services, including travelling longer distances and going to higher-level hospitals for inpatient visits, but also incurred greater medical expenditure along with more inpatient visits, which was in line with the fact that rural patients’ preferences for high-level hospitals and them increasingly choosing to access higher-level hospitals resulted in greater medical expenditures [28,29].

Possible explanations for the effects of TPA policy implementation were as follows. First, traveling costs decreased after implementing TPA policy. The traveling time and expenditure of seeking medical services were considered medical service expenditure [15]. While better transport conditions and better road quality reduced the time expenditure for seeking health care, more choices and increased access to public transport also reduced the travel-related expenditure. Especially for older adults in rural areas, there was sensitive price elasticity due to their low income. Hence, lower transport expenditure meant lower prices of medical services, which stimulated more inpatient visits. Second, TPA policy implementation resulted in more high-level hospital–seeking behaviors. TPA policy implementation made it convenient for older adults in rural areas to go to higher-level hospitals, and patients’ traveling distance increased after policy implementation. Convenient transport changed the “distance decay effect” to a “distance enhancement effect” by removing the geographic barrier and improving patients’ accessibility to medical services, showing that rural patients were more inclined to go to areas with high-quality medical resources at the expense of a longer traveling distance. Additionally, patients were more likely to receive inpatient care in higher-level hospitals after TPA policy implementation. Inequalities in medical resource allocation in China have remained serious [18]. There were 1308 tertiary-level hospitals in China, mainly distributed in central cities. TPA policy implementation accelerated access to high-level medical services and thus increased the medical expenditure. For example, after the opening of high-speed railway, patients were more likely to go to areas with a high density of health care resources [12]. Third, transport increased individual income valuable for seeking inpatient services, which was consistent with previous findings that higher income also was associated with greater access to medical services, and social and economic inequality resulted in health inequality [30,31].

Effect modification analysis revealed that a poor household status, self-rated health status, and age were potential modifiers, and there were modified effects on inpatient expenditure by poor household status, self-rated health status, and age. According to Andersen’s health behavior model, age and self-rated health status were individual predisposing characteristics, and income was an enabling resource; these were closely associated with health care usage. First, inpatient expenditure increased further after TPA policy implementation in poor households. Vulnerable groups are more likely to face barriers when they need health services, including physical overdraft, deficient social security, and low income. In addition, a long traveling time was an additional vulnerability faced by poorer individuals in remote areas when seeking health care [32-35]. However, TPA policy implementation increased their seeking behavior through raising their income. Second, compared to those with a poor self-rated health status, inpatient expenditure was higher among healthy older adults. In contrast with previous findings that self-rated health was an important factor in outpatients’ health care usage [36], we further found that self-rated health status was associated with inpatient care and expenditure. Third, compared with older adults aged >80 years, inpatient expenditure increased further among older adults aged 60-80 years. It could be possible that older adults aged >80 years have a high demand for health care usage due to poor health, regardless of whether the policy was implemented.

Our findings provide new evidence on the association of transport policy implementation with health care–seeking behavior among older adults in rural areas and medical expenditure based on evidence from China. Inspired by the HiAP approach, our findings provide some new implications about improving accessibility and affordability of health services for vulnerable populations in high-income or transitional countries. Especially for rural areas worldwide, in addition to increasing medical resources, improving transport conditions and eliminating barriers to medical service usage are also meaningful measures to promote medical service usage.

### Limitations

This study had several limitations. First, the use of self-reported measures of health care–seeking behavior and medical expenditures might have been underestimated, particularly among older people and those from lower socioeconomic and educational backgrounds, who might be more likely to underreport these factors. Second, the CHARLS questionnaire does not disclose the county-level information of respondents, and its sampling and data collection were beyond our control. As a result, we could not conduct multilevel model analysis and assign the intervention and control groups on the basis of county-level information. But, during the implementation process, TPA policy was actually planned and implemented by provincial governments in a unified manner; hence, using provincial-level information as an identifier for policy intervention could be reliable in this case. Finally, health care–seeking behavior includes a visit rate of twice a week among patients, a nonvisit rate of twice a week, hospitalization rate, and per capita hospital stay. However, these data are not available. Therefore, outpatient and inpatient health care–seeking behavior measures at the individual level were selected to explore their association with TPA policy implementation as microscopic evidence for transport policy and health care usage. Future studies could further explore the complex association between transport policy implementation and other health care–seeking behavior.

### Conclusions

Our findings verify the beneficial effects of TPA policy implementation on inpatient visits and found an increase in medical expenditure among older adults in rural areas. Our findings further provide empirical evidence regarding the relationships of TPA policy implementation with health care–seeking behavior and medical expenditure, which are valuable for addressing difficulties in acquiring health care services and advancing health equality of rural vulnerable groups. A package of policies is further suggested to be integrated into health policies to improve rural patients’ poor access to medical services and enhance their ability to pay for medical expenditure. In addition, concerted efforts are needed to balance the high-quality medical resources and narrow the great gap between urban and rural areas, and finally contribute to healthy ageing and health equity worldwide.

## Appendix

Multimedia Appendix 1 Implementation scope of transport-driven poverty alleviation policy.

Multimedia Appendix 2 Subgroup analysis of TPA policy on medical expenditure among poor- households elderly (yes vs. no).

Multimedia Appendix 3 Outcome distribution of pre-and post-intervention within group from 2011~2018.

Multimedia Appendix 4 Sensitive analysis of rural road mileage on healthcare seeking behavior and medical expenditures among the elderly.

## Abbreviations

CHARLS: China Health and Retirement Longitudinal Study
DID: difference-in-differences
HiAP: Health in All Policies
TPA: Transport-driven Poverty Alleviation

## Data Availability

The original data set is publicly available at the China Health and Retirement Longitudinal Study (CHARLS) website [23]. The data analyzed in our study can be obtained from the corresponding author upon reasonable request.
