# Supplementary material for: Effects of the Implementation of Transport-Driven Poverty Alleviation Policy on Health Care–Seeking Behavior and Medical Expenditure Among Older People in Rural Areas: Quasi-Experimental Study
Source: JMIR Public Health Surveill. 2023 Nov 28;9:e49603. doi: 10.2196/49603 (PMC10716743; doi:10.2196/49603)
Supplement: Multimedia Appendix 1 [file publichealth_v9i1e49603_app1.docx]

**Multimedia Appendix 1**

Implementation scope of transport-driven poverty alleviation policy

| Assignment | Intervention group | Control group |
| --- | --- | --- |
| Provincial administrative region | Hebei, Liaoning, Zhejiang, Fujian, Guangdong, Hainan, Shanxi, Jilin, Heilongjiang, Anhui, Jiangxi, Henan, Hubei, Hunan, Nei Monggol, Guangxi, Chongqing, Sichuan, Guizhou, Yunnan, Tibet, Shaanxi, Gansu, Qinghai, Ningxia, Xinjiang | Shandong, Jiangsu, Beijing, Shanghai, Tianjin |

Key components of China’s Transport-driven poverty alleviation policy

**K1: Promote the external and internal connectivity of backbone channels, and focus on building a “prosperous road” for regional development and poverty alleviation and prosperity.**

• Promote the external and internal connectivity of backbone channels in poverty-stricken areas, promote the convenient and smooth flow of resource elements, involving the upgrading and renovation of 16,000 kilometers of national highways and 46,000 kilometers of ordinary national roads.

**K2: Realize the smooth connection of rural roads to villages and townships, and focus on building a “happy and moderately prosperous road” in poverty-stricken areas.**

• Highlight the principle of “ensuring basic conditions and meeting the bottom line”, and approximately 230,000 kilometers of hardened roads connecting townships and established villages will be constructed. By 2019, we will achieve the comprehensive well-off society goal of establishing hardened roads in established villages that meet the national conditions one year ahead of schedule. Construct approximately 108,000 kilometers of hardened roads connecting villages to solve the problem of unobstructed transportation for large population scale villages, as well as for more than 20 natural villages in ethnic areas.

**K3: Improve the safety level of rural roads and create a “safe and secure road” for people’s travel.**

• A security project for 300,000 kilometers of rural roads will be implemented, dangerous bridges on rural roads will be renovated, the construction of safety and life protection projects on rural roads will be strengthened, and 15,000 dangerous bridges will be renovated. Narrow roads that do not meet the requirements for safe transportation will be reasonably widened and renovated to improve the safety.

**K4: The Road to Wealth.**

• Promote poverty alleviation through “transportation with characteristic industries”, create a “characteristic road to wealth”, and construct approximately 32,000 kilometers of resource roads, tourism roads, and industrial park roads. Further strengthen the basic support role of transportation in industrial poverty alleviation and support the development of characteristic industries in impoverished areas.

**K5: Improve the infrastructure of inland waterway transportation.**

• Further improve the infrastructure of inland waterway transportation, strengthen the renovation and construction of passenger stations in poverty-stricken areas, enhance the depth and service level of rural passenger and freight transportation, and improve the infrastructure of inland waterway transportation. We plan to add and improve 2,600 kilometers of waterway mileage, add 80 new berths, and continue to improve the transportation conditions in rural water network areas.

**K6: Improve transportation services level.**

• Support the transformation and construction of 150 old passenger stations in county towns and 1,100 comprehensive passenger service stations in townships in poverty-stricken areas, and achieve full coverage of established village buses that meet the conditions. Build a rural logistics distribution network at the county, township, and village levels, and promote the development of rural logistics with relevant departments.

.
