# Supplementary material for: Effects of the Implementation of Transport-Driven Poverty Alleviation Policy on Health Care–Seeking Behavior and Medical Expenditure Among Older People in Rural Areas: Quasi-Experimental Study
Source: JMIR Public Health Surveill. 2023 Nov 28;9:e49603. doi: 10.2196/49603 (PMC10716743; doi:10.2196/49603)
Supplement: Multimedia Appendix 2 [file publichealth_v9i1e49603_app2.docx]

**Multimedia Appendix 2**

Subgroup analysis of TPA policy on medical expenditure among poor- households elderly (yes vs. no)

| Poor-households | | | |
| --- | --- | --- | --- |
| Outpatient cost  β(SE) | *P* value | Inpatient cost  β(SE) | *P* value |
| 4.689 | <.001 | .859 | <.001 |
| (0.346) |  | (.227) |  |
| Nonpoor-households | | | |
| Outpatient cost  β(SE) | *P* value | Inpatient cost  β(SE) | *P* value |
| .137 | .855 | 1.263 | .085 |
| (.749) |  | (.728) |  |

Subgroup analysis of TPA policy on medical expenditure among the elderly with different health status

| Bad health status | | | | Good health status | | | |
| --- | --- | --- | --- | --- | --- | --- | --- |
| Outpatient cost  β(SE) | *P* value | Inpatient cost  β(SE) | *P* value | Outpatient cost  β(SE) | *P* value | Inpatient cost  β(SE) | *P* value |
| 4.773 | <.001 | .186 | .42 | 4.364 | <.001 | .648 | <.001 |
| (.354) |  | .232 |  | (.148) |  | (.130) |  |

Subgroup analysis of TPA policy on medical expenditure among the elderly in different age group

| 60~70 | | | |
| --- | --- | --- | --- |
| Outpatient cost  β(SE) | *P* value | Inpatient cost  β(SE) | *P* value |
| 4.310 | <.001 | .584 | <.001 |
| (.196) |  | (.161) |  |

| 70~80 | | | |
| --- | --- | --- | --- |
| Outpatient cost  β(SE) | *P* value | Inpatient cost  β(SE) | *P* value |
| 4.395 | <.001 | .604 | <.001 |
| (.252) |  | (.214) |  |

| >80 | | | |
| --- | --- | --- | --- |
| Outpatient cost  β(SE) | *P* value | Inpatient cost  β(SE) | *P* value |
| 5.045 | <.001 | -.428 | .18 |
| (.350) |  | (.319) |  |
