# Supplementary material for: Effects of the Implementation of Transport-Driven Poverty Alleviation Policy on Health Care–Seeking Behavior and Medical Expenditure Among Older People in Rural Areas: Quasi-Experimental Study
Source: JMIR Public Health Surveill. 2023 Nov 28;9:e49603. doi: 10.2196/49603 (PMC10716743; doi:10.2196/49603)
Supplement: Multimedia Appendix 3 [file publichealth_v9i1e49603_app3.docx]

**Multimedia Appendix 3**

Outcome distribution of pre-and post-intervention within group from 2011~2018

| variables | Total (n=6705) | | | Control group (n=1108) | | | | | | Treatment group (n=5597) | | | | | |
| --- | --- | --- | --- | --- | --- | --- | --- | --- | --- | --- | --- | --- | --- | --- | --- |
|  |  |  |  | Pre | | | Post | | | Pre | | | Post | | |
|  | Mean | SD | Median | Mean | SD | Median | Mean | SD | Median | Mean | SD | Median | Mean | SD | Median |
| Outpatient visits | 2.37 | 2.66 | 1 | 2.25 | 2.61 | 1 | 2.28 | 2.46 | 2 | 2.36 | 2.75 | 1 | 2.36 | 2.97 | 1 |
| Outpatient cost (log) | 5.84 | 1.49 | 5.30 | 6.21 | 1.53 | 5.02 | 6.52 | 1.34 | 5.99 | 5.67 | 1.47 | 5.30 | 6.19 | 1.47 | 6.22 |
| Outpatient cost (yuan) | 1275.32 | 4173.81 | 200 | 1702.42 | 4220.25 | 150 | 1718.04 | 3632.54 | 398 | 1079.22 | 4027.81 | 200 | 1717.92 | 4739.51 | 500 |
| Inpatient visits | 1.57 | 1.34 | 1 | 1.47 | 1.110 | 1 | 1.61 | 1.79 | 1 | 1.53 | 1.24 | 1 | 1.70 | 1.52 | 1 |
| Inpatient cost (log) | 9.20 | 1.28 | 9.21 | 9.72 | 1.14 | 8.99 | 9.82 | 1.31 | 9.21 | 8.96 | 1.27 | 9.90 | 9.39 | 1.23 | 9.90 |
| Inpatient cost (yuan) | 21088.21 | 19449.01 | 10000 | 23133.35 | 17273.55 | 8000 | 26138.87 | 33196.33 | 10000 | 19766.56 | 14894.72 | 20000 | 22291.7 | 24787.45 | 20000 |
