# Supplementary material for: Effects of the Implementation of Transport-Driven Poverty Alleviation Policy on Health Care–Seeking Behavior and Medical Expenditure Among Older People in Rural Areas: Quasi-Experimental Study
Source: JMIR Public Health Surveill. 2023 Nov 28;9:e49603. doi: 10.2196/49603 (PMC10716743; doi:10.2196/49603)
Supplement: Multimedia Appendix 4 [file publichealth_v9i1e49603_app4.docx]

**Multimedia Appendix 4**

Sensitive analysis of rural road mileage on healthcare seeking behavior and medical expenditures among the elderly

| Outpatient times  β(SE) | *P* value | Outpatient cost  β(SE) | *P* value |
| --- | --- | --- | --- |
| .096 | .46 | .810 | <.001 |
| (.130) |  | (.081) |  |
|  |  |  |  |
| Inpatient times  β(SE) | *P* value | Inpatient cost  β(SE) | *P* value |
| .266 | <.001 | .089 | <.001 |
| (.075) |  | (.008) |  |

Sensitive analysis of excluding effect of health insurance and pensions among the elderly

| Medicine cost  β(SE) | *P* value | Outpatient cost  β(SE) | *P* value | Inpatient cost  β(SE) | *P* value |
| --- | --- | --- | --- | --- | --- |
| .404 | <.001 | 4.941 | <.001 | .852 | <.001 |
| (.043) |  | (.241) |  | (.196) |  |

Sensitive analysis by PSM-DID of TPA policy on medical expenditures among the elderly

| Outpatient cost (ln)  β(SE) | *P* value | Inpatient cost (ln)  β(SE) | *P* value |
| --- | --- | --- | --- |
| 2.996 | <.001 | .618 | <.001 |
| (.559) |  | (.119) |  |

Sensitive analysis of Placebo test by setting policy time to 2015

| Outpatient times  β(SE) | *P* value | Outpatient cost  β(SE) | *P* value |
| --- | --- | --- | --- |
| .241 | .45 | -.206 | .44 |
| (.316) |  | (.268) |  |
| Inpatient times  β(SE) | *P* value | Inpatient cost  β(SE) | *P* value |
| .032 | .86 | .412 | .23 |
| (.183) |  | (.338) |  |
